# Diagnostic Evaluation and Management of Idiopathic Chylous Ascites: A Report of a Surgical Case

**DOI:** 10.7759/cureus.83562

**Published:** 2025-05-06

**Authors:** Ann Wu, Roneil Parikh, Laurence Gluch

**Affiliations:** 1 Breast and Endocrine Surgery, Concord Repatriation General Hospital, Sydney, AUS

**Keywords:** ascites of unexplained origin, case report, chylous ascites, lymphatic hypertension, lymphatic leak, milky ascites

## Abstract

Chylous ascites (CA) is the pathological accumulation of lymphatic fluid in the peritoneal cavity. The ascitic fluid has a high concentration of triglycerides and chylomicrons, giving it a milky appearance. Owing to its infrequent diagnosis, there is limited literature describing the workup and treatment of idiopathic chylous ascites (ICA). This report describes the case of a 39-year-old woman who presented with undifferentiated abdominal pain, with the investigations leading to a diagnosis of ICA and interventions which led to complete resolution of symptoms over a follow-up period of eight months. We propose a systematic method to exclude alternative causes of chyle leak, thus aiding in the diagnosis of ICA.

## Introduction

Chylous ascites (CA) is a rare form of ascites characterised by exudation of lipid-rich lymph into the peritoneal cavity [1]. In adults, CA occurs when there is an acquired disruption or obstruction of lymphatic vasculature, the most common causes of which are malignancy, infection, and trauma [2]. CA is an uncommon cause of undifferentiated abdominal pain, with an estimated incidence of one in 20,000 [3]. Idiopathic chylous ascites (ICA) is rarer still, with limited evidence on diagnosis and management. The diagnosis of ICA requires systematic exclusion of alternative pathologies.

In this case report, we outline our experience of diagnosing and treating ICA at an Australian tertiary-level hospital. The causes, the assessment, investigations, and the management of CA were reviewed. The definitive aetiology of CA in this case could not be ascertained; however, the possibility of lymphatic obstruction due to transient mesenteric volvulus is discussed.

## Case presentation

A 39-year-old woman presented to the emergency department with a three-day history of lower abdominal pain. This was associated with bloating, nausea, and vomiting, but with normal bowel habitus. Apart from treatment for childhood tuberculosis in China, she had no significant past medical history, prior abdominal surgeries, or abdominal trauma. On presentation, she had a low-grade fever of 37.6°C but was haemodynamically stable. Examination revealed a mildly distended abdomen with marked tenderness, rebound, and peritonism in the left hypochondrium and epigastric regions. Her white cell count, C-reactive protein, and lipase were mildly elevated. Liver function enzymes were unremarkable (Table 1).

**Table 1:** Pathology results on admission

Result	Patient Value	Reference Range
White cell count	16.7 x 10^9^/L	4.0 - 10.0 x 10^9^/L
Haemoglobin	145 g/L	120 - 150 g/L
Platelets	195 x 10^9^/L	150 - 400 x 10^9^/L
Neutrophils	15.3 x 10^9^/L	2.0 - 7.0 x 10^9^/L
Lymphocytes	0.8 x 10^9^/L	1.0 - 3.0 x 10^9^/L
Albumin	39 g/L	31 - 47 g/L
Bilirubin	16 µmol/L	<20 µmol/L
Alkaline phosphatase	64 U/L	30 - 110 U/L
Alanine transaminase	10 U/L	5-35
Aspartate aminotransferase	19 U/L	10 - 35 U/L
Gamma-glutamyl transferase	24 U/L	10 - 35 U/L
Lipase	144 U/L	10 - 60 U/L
C-reactive protein	11.6 mg/L	<5 mg/L

**Figure 1:**
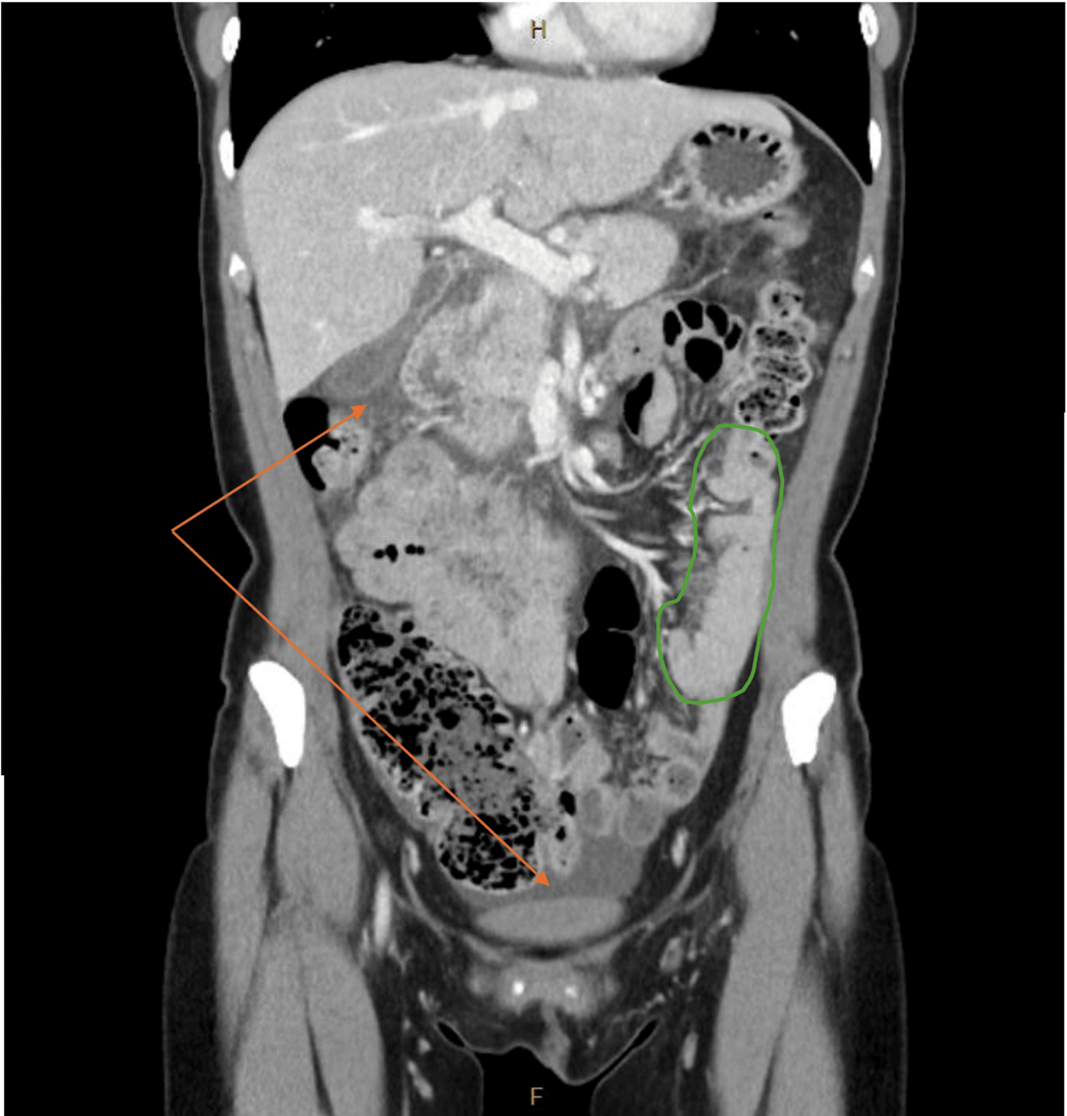
CT of the abdomen and pelvis (coronal view) in portal venous phase demonstrating cluster of displaced small bowel (outlined in green) and intraperitoneal free fluid (orange arrows)

A computed tomography (CT) scan of the abdomen and pelvis suggested an unusual appearance of displaced small bowel to the left upper quadrant, twisted mesentery, and a moderate volume of free fluid. There was normal mesenteric venous enhancement and no mural oedema, obstruction, or perforation. There was no evidence of lymphadenopathy, cirrhosis, steatosis, or portal venous distension (Figure 1).

**Figure 2:**
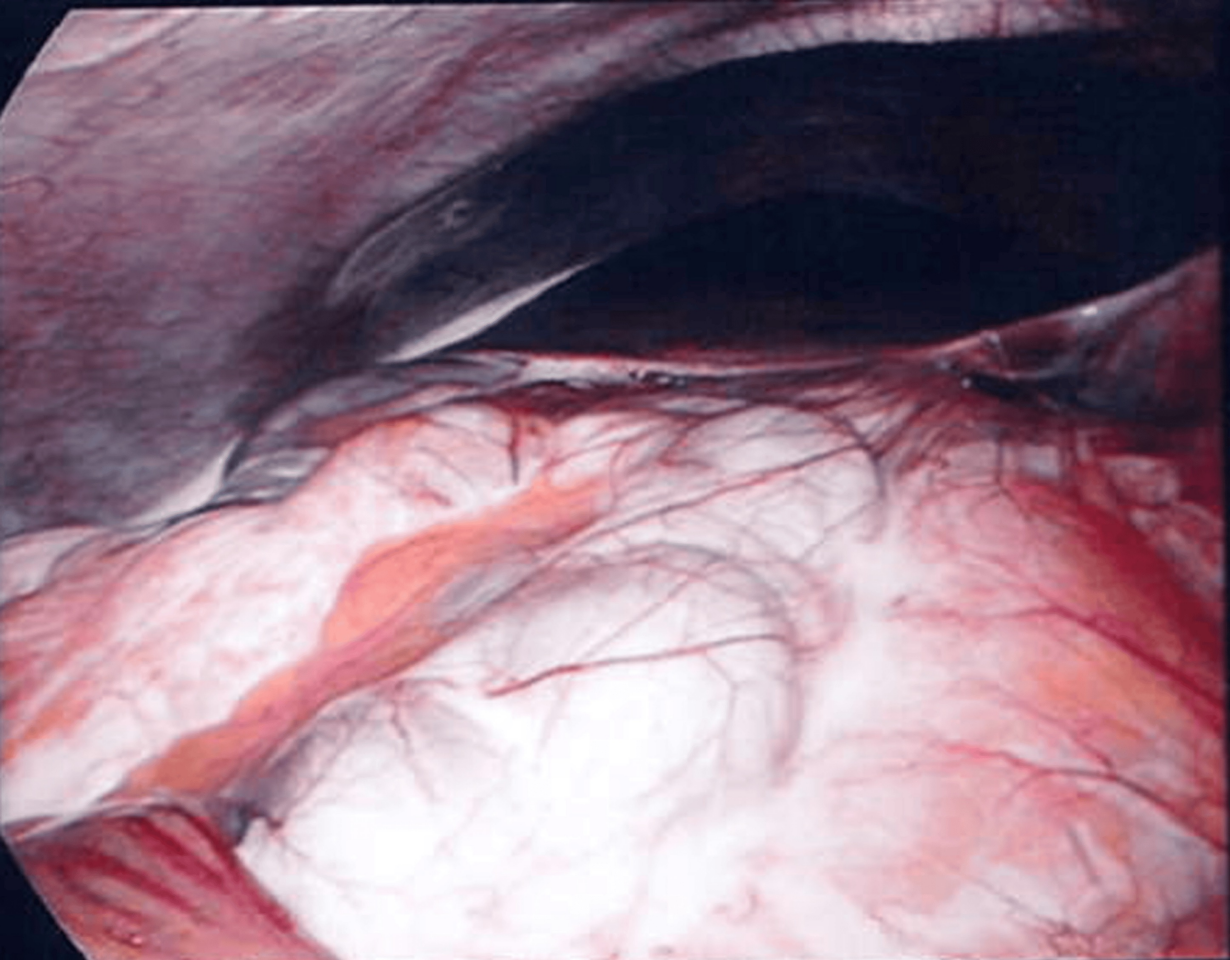
Diagnostic laparoscopy looking towards the right upper quadrant. The liver and hepatic flexure are in view, coated by a white sheen of chylous ascites.

**Figure 3:**
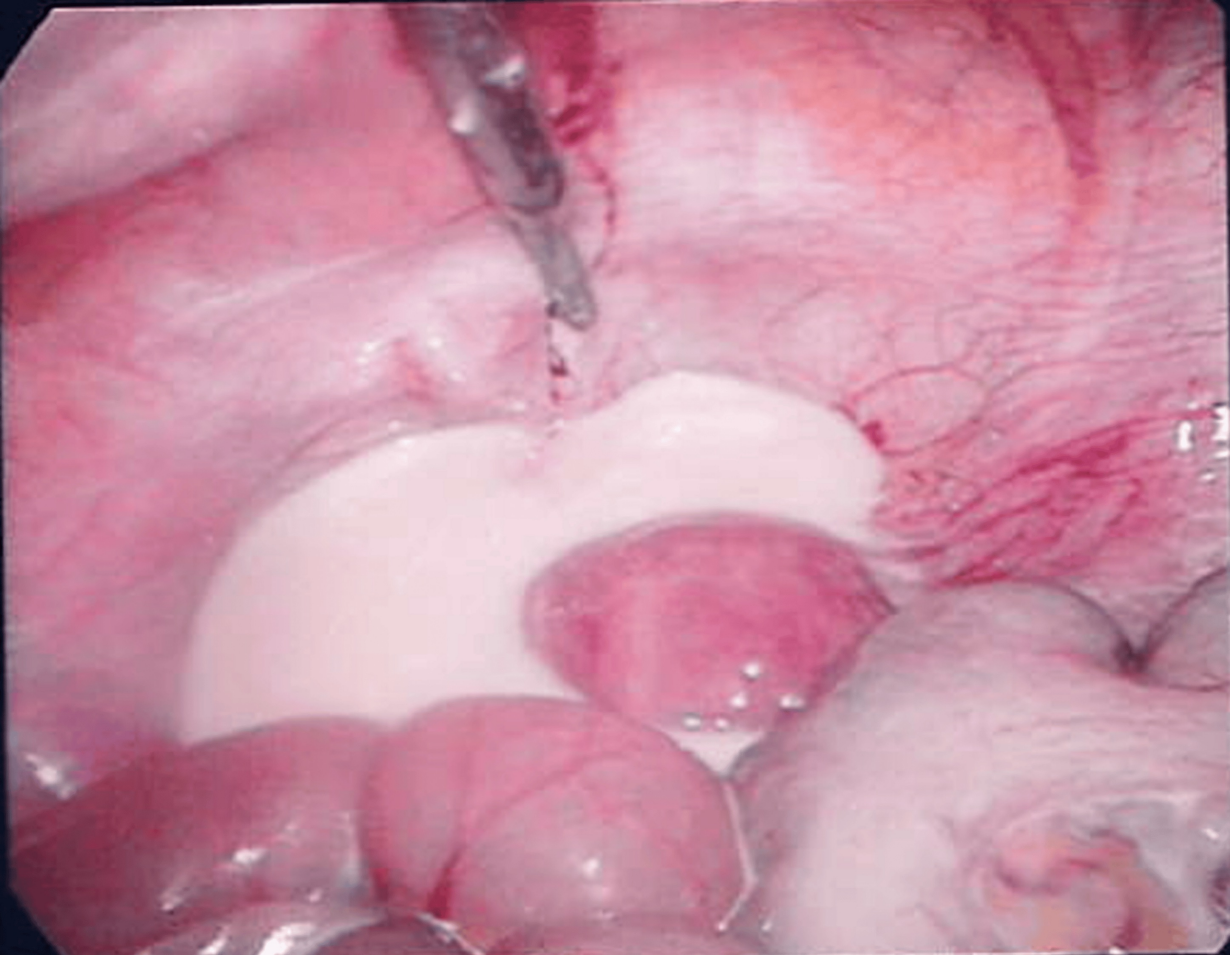
Diagnostic laparoscopy looking towards the pelvis shows accumulation of chylous ascites.

Given her examination findings, the patient proceeded to diagnostic laparoscopy, which uncovered four-quadrant CA (Figures 2-3), most pronounced at the transverse mesocolon. Examination of the abdomen and pelvis, including the entirety of the small bowel and mesentery, did not reveal any clear obstruction. Following aspiration of fluid and a drain insertion, the abdomen was closed. Postoperatively, she was fasted, received total parenteral nutrition (TPN), and completed a course of empiric intravenous antibiotics. Regular dietician review, daily liver function enzyme and serum electrolyte levels, blood glucose monitoring, and daily weights were performed to prevent and monitor for potential side effects of TPN administration. 

Biochemical analysis of the ascitic fluid demonstrated elevated triglycerides and the presence of chylomicrons, confirming the diagnosis of CA (Table 2). The serum ascites albumin gradient (SAAG) was initially high; however, this was difficult to ascertain due to a significant change in serum albumin from 39g/L (SAAG 2.2g/dL) on the day of presentation to 22g/L (SAAG 0.5g/dL) following laparoscopy. A multidisciplinary input from infectious diseases, haematology, and gastroenterology specialities was sought to aid in ascertaining the aetiology of her acute CA.

**Table 2:** Ascitic fluid biochemistry

Result	Patient Value	Reference Range
Albumin	17 g/L	<30g/L
Amylase	115 U/L	18-64 U/L
Glucose	3.6 mmol/L	7-10 mmol/L
Protein	27 g/L	<30g/L
Triglycerides	27.9 mmol/L	<1.69 mmol/L
Lipid electrophoresis	Chylomicron positive	Negative

The ascitic fluid was negative for cytology, microscopy, and culture. Tuberculosis was not detected in serum or ascitic fluid. Serologies were performed to investigate potential causes of lymphatic obstruction or fibrosis, including schistosomiasis, human immunodeficiency virus, hepatitis B and C, immunoglobulin G4, and serum angiotensin converting enzyme levels, all of which were unremarkable.

Tumour markers (cancer antigen 125, cancer antigen 19-9, and carcinoembryonic antigen) were likewise within normal limits. Lymphoproliferative disease was excluded following investigation with serum lactate dehydrogenase, haptoglobin, immunoglobulin G/A/M levels, serum protein electrophoresis and immunoelectrophoresis, serum free light chains, peripheral flow cytometry, and beta-2 microglobulin. Stool samples were negative for parasitic, bacterial, and viral pathogens.

Lymphangiography and CT lymphangiography were performed on day two postoperatively. No leakage of lymph in the chest, abdomen (including at the cisterna chyli), retroperitoneum, or pelvis was noted.

Following the reduction in drain output, oral intake was increased in a stepwise manner until a low-fat diet was tolerated on hospital day 11, at which point TPN was ceased. She was discharged home on day 15 with advice to continue the low-fat diet, and short courses of oral amoxicillin-clavulanic acid and pantoprazole.

Three months after discharge, a fluorodeoxyglucose (FDG)-positron emission tomography (PET) did not demonstrate any uptake to raise concern for an infectious or malignant process. When reviewed in the outpatient clinic, the patient reported complete resolution of all symptoms, tolerance of a full-fat diet, and was participating in work and activities of daily living without restrictions. Eight months following discharge, she remained asymptomatic with normal liver function tests. No long-term pharmacotherapy was prescribed at any point. An ultrasound with liver elastography was arranged, but she was subsequently lost to follow-up.

Despite extensive investigation, no cause for the acute, atraumatic CA was determined, leading to a diagnosis of ICA.

## Discussion

Intraperitoneal accumulation of milky, triglyceride-rich lymphatic fluid is the defining characteristic of CA [4]. Patients may present with non-specific symptoms of abdominal distension, malabsorption, abdominal pain, or peripheral oedema [2]. Idiopathic acute chylous peritonitis is a rare condition, the incidence of which is unknown. The incidence of all cause CA is estimated to be one in 20,000 [5].

It is estimated that over half of the body’s circulating protein passes through lymph each day; the abdominal viscera, in turn, contribute to over half of the body’s total lymph content. The gut lymphatics are thus critical in maintaining homeostasis of plasma volume and composition [1].

Two pathophysiological mechanisms are commonly described in the development of CA: (i) lymphatic hypertension within the bowel or mesentery, which may arise from fibrosis, obstruction, or portal hypertension, and (ii) leakage from abnormal lymphatic vessels via lymphoperitoneal fistula or retroperitoneal megalymphatics [2].

**Figure 4:**
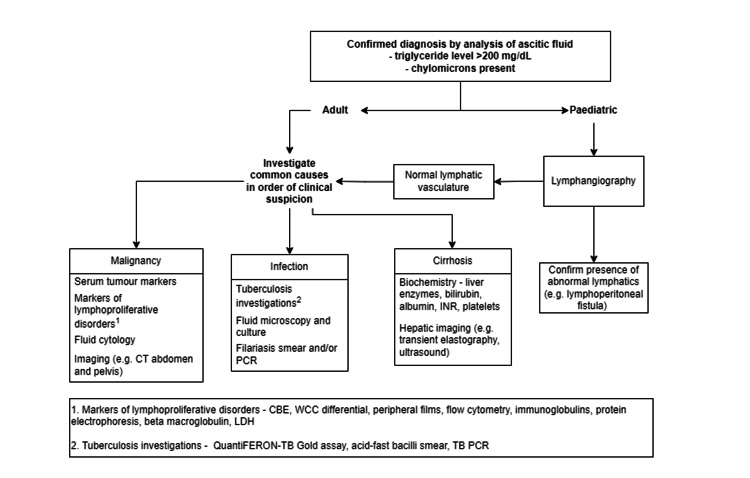
Recommended investigations for patients presenting with atraumatic chylous ascites Systematic investigation is recommended to determine the aetiology of atraumatic chylous ascites. For adults or children without evidence of lymphovascular abnormality, the presence of malignancy, infection, or hepatic cirrhosis should be reviewed in order of clinical suspicion. CT: computed tomography; PCR: polymerase chain reaction; INR: international normalised ratio; CBE: complete blood exam; WCC: white cell count; LDH: lactate dehydrogenase; TB: tuberculosis Image credits: Ann Wu (Author)

In adults, the most common aetiologies of CA are acquired. Malignancy, cirrhosis, and infection account for over two-thirds of cases [6]. Oosterbach et al. report that up to 85% of chylous effusions are secondary to lymphoma [7]. Of the infectious causes, tuberculosis and filariasis are by far the most common and should be strongly suspected in patients with a history of travel to endemic regions [1]. One study reports a postoperative incidence of 7.4% after tumour resection in the retroperitoneum and upper abdomen [8]. In the paediatric cohort, congenital abnormality of lymphatic vessels is the leading cause [3]. Investigation for a causative pathology should be undertaken thoroughly before a diagnosis of ICA is made (Figure 4) [3].

In the current case, following the intraoperative finding of CA, we undertook a systematic approach to ascertain the cause. Firstly, the diagnosis of CA was confirmed by analysis of triglyceride and chylomicron levels. The presence of portal hypertension was investigated by the serum-ascites albumin gradient and portal vein calibre on imaging. Lymphangiography was then performed to look for lymphatic extravasation. Given the ongoing diagnostic uncertainty, screening for underlying malignancy and infection was also performed by flow cytometry, peripheral blood smear, tumour markers, serological markers of lymphoproliferative disorders, and FDG-PET.

We postulate that the displaced bowel and mesenteric swirling, as seen on the preoperative CT, induced a state of transient obstruction within the mesenteric lymphatics, causing lymphatic hypertension and chyle leak. The serum albumin ascites gradient was suggestive of a high hepatic pressure gradient, as seen in portal hypertension. This could be mimicked by the high-pressure system of the twisted mesentery, resulting in ascites of a transudative origin. The initial SAAG elevation and development of hypoalbuminaemia at post-laparoscopy day 1, followed by a gradual return to the reference range, correlates with the loss of protein during drainage of the ascitic fluid. Additionally, this is supported by the post-operative lymphangiogram, which did not demonstrate an anatomical cause for chyle leak. 

In the absence of an underlying cause, the mainstays of treatment are drainage and nutritional support [9]. Operative exploration by means of laparoscopy or laparotomy may be necessary for the acutely unwell patient with diagnostic uncertainty, as seen in this case. Paracentesis is an alternative means of drainage which may be considered [6].

To minimise chyle production, oral lipids are reduced or eliminated from the diet by a low-fat diet, supplemental medium-chain fatty acids, or total parenteral nutrition. Due to their absorption into intestinal lymphatics as chylomicrons, the avoidance of long-chain fatty acids is of particular importance [10]. By contrast, medium-chain fatty acids are absorbed by enterocytes and pass to the liver via the portal vein, and therefore do not contribute to the formation of chyle [9]. The adjunct use of orlistat to decrease fat absorption can also be considered. In cases that are refractory to dietary adjustments, octreotide may be trialled [4].

CA, which proves refractory to dietary measures, may be treated with lymphangiography and lipiodol embolisation by an interventional radiologist [11] or surgical intervention by primary repair or placement of peritoneovenous shunt [8]. Lymphangiography can be used to embolise the culprit lymphatic channel or as an adjunct to assist with intra-operative localisation of the suspected lymphatic disruption [12]. The rate of locating lymphatic leak and successful embolisation is reported to be as high as 79% and 71%, respectively [13]. The success rates of lymphangiography or primary repair have not been documented for ICA. 

## Conclusions

Accumulation of milky-white fluid in the peritoneal cavity should raise strong suspicion of CA, the hallmark of which is elevated triglycerides and chylomicrons. In children and postoperative patients, CA is commonly due to structural abnormalities of the lymphatic vasculature. ICA is an uncommon pathology that should be treated as a diagnosis of exclusion. Thorough assessment should be directed at the exclusion of underlying malignancy, hepatic cirrhosis, or infection in adults. This must include biochemistry. microscopy, and culture of the ascitic fluid.

Management of CA is directed at maximising ascitic drainage and minimising chyle production. Modifying the oral diet to eliminate long-chain fatty acids is an essential step here. Surgical intervention or embolisation should be considered in cases that fail to improve through diet and ascitic drainage. In the case of acute chylous peritonitis reported, no cause was determined despite systemic assessment. The patient’s symptoms resolved with drainage and total parenteral nutrition followed by a low-fat diet, and she remained well at follow-up eight months later.
